# Bilateral healing of pseudofractures in hypophosphatasia with teriparatide: a case report

**DOI:** 10.1186/s13256-026-05903-5

**Published:** 2026-03-24

**Authors:** Elaf Al-Saoudi, Nicola Hepp, Jens-Erik Beck Jensen

**Affiliations:** 1 Department of Endocrinology, Hvidovre Hospital, Copenhagen University Hospital, Kettegård Alle 30, 2650 Hvidovre, Denmark; 2 Department of Medicine, Herlev Hospital, Copenhagen University Hospital, Borgmester Ib Juuls Vej 1, 2730 Herlev, Denmark; 3 Department of Clinical Genetics, Rigshospitalet, Copenhagen University Hospital, Blegdamsvej 9, 2100 Copenhagen, Denmark

**Keywords:** Hypophosphatasia, Teriparatide, Fracture healing, Pseudofractures

## Abstract

**Background:**

Hypophosphatasia is a rare, inherited metabolic bone disease characterized by impaired activity of tissue-nonspecific alkaline phosphatase leading to pathological mineralization of hard tissue, predisposing to poorly healing fractures. Limited access to enzyme replacement therapy makes the treatment of adults with hypophosphatasia challenging. A few case reports have described a beneficial effect of teriparatide on fracture healing in hypophosphatasia. However, this is the first case of a male patient with pediatric-onset hypophosphatasia, not previously exposed to antiresorptive therapy, treated with teriparatide for bilateral femoral pseudofractures.

**Case presentation:**

We report a 59-old white man with pediatric-onset hypophosphatasia who developed bilateral femoral pseudofractures that were successfully treated with teriparatide. At the age of 54 years, our patient complained about reduced physical activity and pain in both hips and femoral bones. X-ray and later computed tomography scan revealed bilateral femoral pseudofractures. Dual energy X-ray absorptiometry showed normal bone mineral density. Not previously exposed to antiresorptive treatment, our patient was treated with conventional doses of teriparatide (Forsteo^®^, 20 µg/day, subcutaneous) for 18 months. Markers of bone formation and bone resorption increased twofold at 3 months of treatment. At 6 months of treatment with teriparatide normal levels of alkaline phosphatase as well as pyridoxal 5′-phosphate were measured. Early signs of fracture healing were observed at 6 months, and after 18 months of teriparatide treatment, the complete healing of both femoral pseudofractures was proven by X-ray and a computed tomography scan.

**Conclusion:**

Treatment with teriparatide induces beneficial clinical, biochemical, and radiological responses and may provide an efficient treatment option for adults with hypophosphatasia and pseudofractures or poor healing fractures.

## Background

Hypophosphatasia (HPP) is a rare, inherited metabolic bone disease caused by pathogenic variants in *ALPL*, leading to reduced activity of tissue-nonspecific alkaline phosphatase (TNSALP) and pathological bone mineralization [1]. The disease can be autosomal dominant or recessive and leads to reduced levels of alkaline phosphatase (ALP) and elevated pyridoxal 5′-phosphate (PLP) [1–3].

HPP is a multisystemic disease with broad-ranging clinical presentations and highly variable severity, and more than 450 different variants in *ALPL* have been reported [1, 4, 5]. The clinical features in adults with HPP range from unspecific symptoms such as muscle pain and weakness, headaches, and depression to recurrent, low-energy and poorly healing fractures typically involving the metatarsals [1]. Patients with pediatric-onset of symptoms and biallelic pathogenic variants in *ALPL* are often more severely affected and at risk of developing femoral pseudofractures located on the lateral aspect of the proximal femurs [6].

However, fracture treatment in adults with HPP is challenging. Targeted enzyme replacement therapy with asfotase alfa (AA) is currently only approved for patients with pediatric-onset HPP and may improve fracture healing in adults with HPP [7, 8]. However, the access to AA-therapy is limited owing to its prohibitive costs and is not available for patients with adult-onset of thedisease. In addition, the treatment of fractures and low bone mineral density (BMD) in HPP with antiresorptive agents is not recommended as it is hypothesized that these agents may further suppress TNSALP activity as well as bone remodeling, leading to atypical femoral fractures (AFF) [1]. Bone-forming agents such as recombinant human parathyroid hormone (PTH) 1–34 (that is, teriparatide) may be an alternative treatment option and have been used off-label in several patients with HPP and fractures [9–14]. In several of these cases, improved bone pain, healed stress, and pseudofractures due to teriparatide treatment have been described [9, 12–14].

Here, we report a 59-year-old man with pediatric-onset HPP who presented with bilateral pseudofractures that healed completely after 18 months of teriparatide treatment.

## Case presentation

Our patient, a white man, was born as the fourth child to nonconsanguineous parents. The patient was diagnosed with HPP at the age of 2 years owing to mild clinical symptoms and persistently low levels of ALP in serum as well as elevated phosphoethanolamine (PEA) in the urine. Developmental milestones have not been registered, but the patient reported that he had delayed motor development and has always had gait difficulties. In childhood, a unilateral femoral fracture due to a low energy trauma was reported. In adulthood, genetic testing was performed, and the patient was found compound heterozygous for two pathogenic variants in *ALPL*: c.571G > A, p.(Glu191Lys) and c.512A > G, p.(His171Arg).

**Fig. 1:**
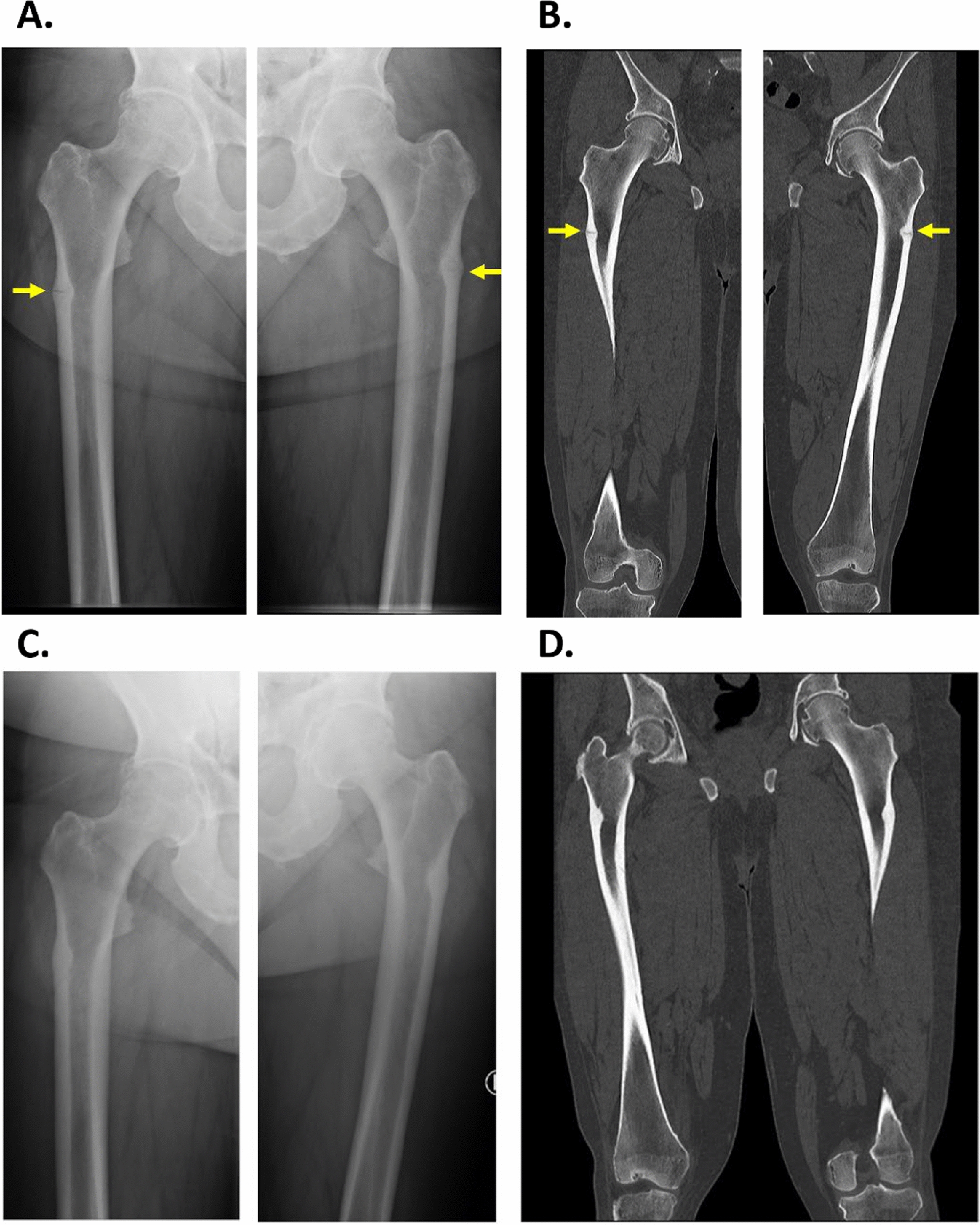
Results of X-ray and computed tomography scan controls. X-ray (**A**) and computed tomography scan (**B**) performed before treatment with teriparatide showed pseudofractures on the lateral aspects of the proximal femora bilaterally (yellow arrows). X-ray (**C**) and computed tomography scan (**D**) performed 17 months after treatment showed complete healing of both femoral pseudofractures

At the age of 54 years, the patient participated in a clinical trial at our endocrinological outpatient clinic. At that time, he complained about worsening of physical ability with reduced walking capacity for several years and muscle weakness of the lower extremity. Further, he reported chronic pain in the hips, knees, and feet as well as dental manifestations including periodontitis, loss of permanent teeth, and loose teeth. We performed an X-ray to evaluate the reason for pain and reduced physical ability. The X-ray and subsequent computed tomography (CT) scan of the hip and femurs revealed pseudofractures on the lateral aspects of the proximal femora bilaterally (Fig. 1). In addition, the X-ray of the feet showed sequelae after a nondisplaced transverse fracture in the proximal half of the fifth metatarsal of the left foot. Dual-energy X-ray absorptiometry showed normal BMD (Table 1).

**Table 1:** Data of the case patient at baseline, during, and after treatment with teriparatide

Measurements	Months of treatment						After treatment
Biochemical parameters	0	1	2	3	6	18	24 (months)
p-ALP (35–105 U/L)	< 10	16	13	14	39	13	< 10
p-PLP (20–121 nmol/L)	> 1000	988	1100	868	6.0	–	–
p-Phosphate (0.76–1.41 mmol/L)	1.23	1.43	1.46	1.53	1.62	1.38	1.38
p-Calcium ion (1.18–1.32 mmol/L)	1.21	1.25	1.25	1.20	1.28	1.13	1.19
25-OH-vitamin D (> 50 nmol/L)	58	80	79	81	62	64	58
p-PTH (1.1–7.1 pmol/L)	4.5	3.6	3.8	3.4	1.9	2.1	8.1
s-BALP (µg/L)	< 1	1.9	2.1	1.3	> 1.0	1.7	–
s-Osteocalcin (µg/L)	10.6	24.0	31.5	35.4	13.4	16.4	–
s-PINP (µg/L)	37.1	99.6	76.6	80.4	63.1	62.3	32.7
s-βCTX (ng/L)	124	103	194	246	42.7	66.5	88,9
Physical ability							
6-MWT (distance in m)	361.6	–	–	–	272.2	254.30	–
30-s CST (number of stands)	6	–	–	–	6	8	–
T-scores (DXA)							
Spine (L1–L4)	+2.7	–	–	–	–	–	+4.3
Total hip (g/cm^2^)	+1.3	–	–	–	–	–	+3.4
Femoral neck (g/cm^2^)	+2.4						+ 4.7

ALP, alkaline phosphatase; PLP, pyridoxal 5′-phosphate; 25-OH-vitamin D, 25-hydroxy-vitamin D; PTH, parathyroid hormone; CTX, C-terminal telopeptide of type I collagen; P1NP, procollagen type I N-terminal propeptide; BALP, bone-specific alkaline phosphatase; 6-MWT, 6-minute walk test; 30-s CST, 30-s chair stand test

s-, serum

p-, plasma

Owing to no surgical treatment options from the orthopedic surgeons for the bilateral pseudofractures, and a subsequent denial of AA treatment by the Regional Drug Committee, treatment with teriparatide, Forsteo^®^ 20 µg/d was initiated when the patient was 57 years old. Before treatment, a height of 171.1 cm, a weight of 122.5 kg and a head circumference of 61.5 cm were registered. The treatment was well tolerated, and the patient reported no side effects. X-rays of the femora after 3 months of treatment showed no signs of fracture healing. After 6 months, X-ray controls showed early stages of fracture healing. Teriparatide treatment was stopped after 18 months owing to complete healing of both femoral pseudofractures, confirmed by an X-ray and CT scan (Fig. 1).

Biochemical parameters are presented in Table 1. Bone turnover markers, plasma procollagen type I N-terminal propeptide (P1NP) and C-terminal telopeptide of type I collagen (CTX) increased twofold at 3 months of treatment and decreased afterwards. ALP increased to a normal measurement at 6 months but was measured beneath the normal range later again. PLP normalized as well at 6 months. BMD increased substantially owing to anabolic treatment (Table 1). The patient reported subjective improvement of pain at the hips and femoral bones as well as better physical ability. However, the 6-minute walk test (6-MWT) did not show improvement, while the ability to raise from a sitting position (30 second chair stand test) ameliorated after treatment (Table 1). At 3 years after the discontinuation of teriparatide treatment, the patient remained free of pain and fractures.

## Discussion and conclusion

To the best of our knowledge, this is the first case of an adult male patient with recessive pediatric-onset HPP who has not been exposed to antiresorptive agents and developed bilateral femoral pseudofractures that healed after conventional daily doses of teriparatide for 18 months.

Previously, a few case reports have described a beneficial effect of teriparatide on the fracture healing of femoral, tibial and metatarsal stress fractures in adults with HPP [9, 10, 13, 15]. In comparison with our case, several of these patients were treated with bisphosphonates prior to teriparatide [11, 16]. To our knowledge, only one male and three female patients with adult-onset HPP who received teriparatide without prior bisphosphonate treatment have been described [10, 15, 17].

The first case described was a 56-year-old woman, heterozygous for a pathogenic variant in *ALPL* who was treated with teriparatide for 18 months due to slowly healing metatarsal stress fractures and a spontaneous proximal femoral fracture. This patient showed normal levels of ALP, and increasing bone turnover markers during treatment as well as radiographically healed femoral fracture and metatarsal fractures within 2–4 months [15]. Likewise, sustained biochemical response to teriparatide was seen in a 48-year-old male patient with HPP. This patient, also heterozygous for a pathogenic variant in *ALPL* received teriparatide owing to delayed healing of a low energy fracture at the distal tibia and was only treated for 12 weeks as the CT scan showed significant induction of bone callus formation at that time [14]. Compared with these cases, the later healing of the fractures and the decreasing levels of ALP after 6 months in our patient could be explained by the more severely reduced ALP activity due to the compound heterozygosity of pathogenic variants in *ALPL* as well as the longer treatment duration.

Further, the case of a 53-year old woman with HPP (compound heterozygous for pathogenic variants in *ALPL*) and femoral pseudofractures that were treated with teriparatide for 18 months has been reported [10]. In contrast to our case, fracture healing was already achieved after 8 months of treatment. A bone biopsy, before and 5 months after teriparatide treatment, revealed increased amounts of osteoblasts numbers [10]. However, biochemical response to teriparatide was not sustained as serum ALP returned to baseline between 8 and 13 months of treatment, which consistent with our observations [10].

The effect of teriparatide treatment in our patient and other patients may be explained by the presence of alleles preserving residual enzymatic activity and thereby the ability to upregulate TNSALP expression in osteoblasts. The most frequent *ALPL* variant in white individuals (c.571G > A, p.(Glu191Lys)), also presented in our patient, was reported to have 88% of TNSALP wild-type activity in vitro and is often found in adults with mild forms of HPP [18]. However, patients more severely affected and compound heterozygous for pathogenic variants in *ALPL* seem to respond slower to teriparatide treatment in case of fracture healing, and ALP levels seem to decrease again after longer treatment durations. Therefore, we speculate that patients with severe HPP may need longer treatment durations. In addition, the massive increase in BMD in both lumbar spine and hip in our patient indicate that teriparatide not only adds bone collagen but also helps to mineralize the bone. However, teriparatide treatment duration is limited to only 2 years. By contrast, it has been suggested that teriparatide administration can be prolonged to 4 years of treatment with less frequent doses than conventional daily doses and still have clinical effect [13]. Thus, alongside *ALPL* pathogenic variants and ALP residual activity, PTH analog treatment may be most beneficial in patients with HPP who have a mild or moderate phenotype. In addition, it is important to monitor potential adverse effects of teriparatide, such as bone pain, anemia, elevated cholesterol, as well as nausea, fatigue, dizziness, and headache, which are often related to hypercalcemia. Notably, our patient did not experience any adverse effects during treatment.

New anabolic agents that work via the reduction of sclerostin are, owing to their dual-mode action with reduced resorption and increased formation, probably not better than teriparatide, although casuistic presentations have found fracture healing in patients with HPP without an increase in ALP [19]. Given the limited availability of the enzyme replacement therapy with AA, treatment with anabolic agents is, however, necessary to improve fracture healing and bone mineralization in adults with HPP.

In conclusion, we present a case of 59-year-old man with pediatric-onset HPP and bilateral femoral pseudofractures, not previously exposed to antiresorptive therapy, treated with conventional daily doses of teriparatide for 18 months. Treatment with teriparatide induces beneficial clinical, biochemical, and radiological responses and may represent an efficient treatment option for adults with HPP and pseudofractures or poor healing fractures. However, response to treatment may vary depending on the genotype and residual ALP activity.

## Data Availability

The case report is based on information from the patient’s electronic health records.

## Abbreviations

AA: Asfotase alfa
ALP: Alkaline phosphatase
BALP: Bone-specific alkaline phosphatase
BMD: Bone mineral density
CT: Computed tomography
CTX: C-terminal telopeptide of type I collagen
DXA: Dual energy X-ray absorptiometry
HPP: Hypophosphatasia
PEA: Phosphoethanolamine
PLP: Pyridoxal 5′-phosphate
PTH: Parathyroid hormone
P1NP: Procollagen type I N-terminal propetide
TNSALP: Tissue-nonspecific alkaline phosphatase
6-MWT: 6-minute walk test
30-s CST: 30-second chair stand test
25-OH-vitamin D: 25-Hydroxy-vitamin D
